# Supplementary material for: Causal Relationship between Aging and Anorexia Nervosa: A White-Matter-Microstructure-Mediated Mendelian Randomization Analysis
Source: Biomedicines. 2024 Aug 16;12(8):1874. doi: 10.3390/biomedicines12081874 (PMC11351342; doi:10.3390/biomedicines12081874)
Supplement: Supplementary file 1 [file biomedicines-12-01874-s001.zip › Supplementary Figure/Figure S4/Singe SNP plot of MR analysis for TL to ubm-b-1573.pdf]

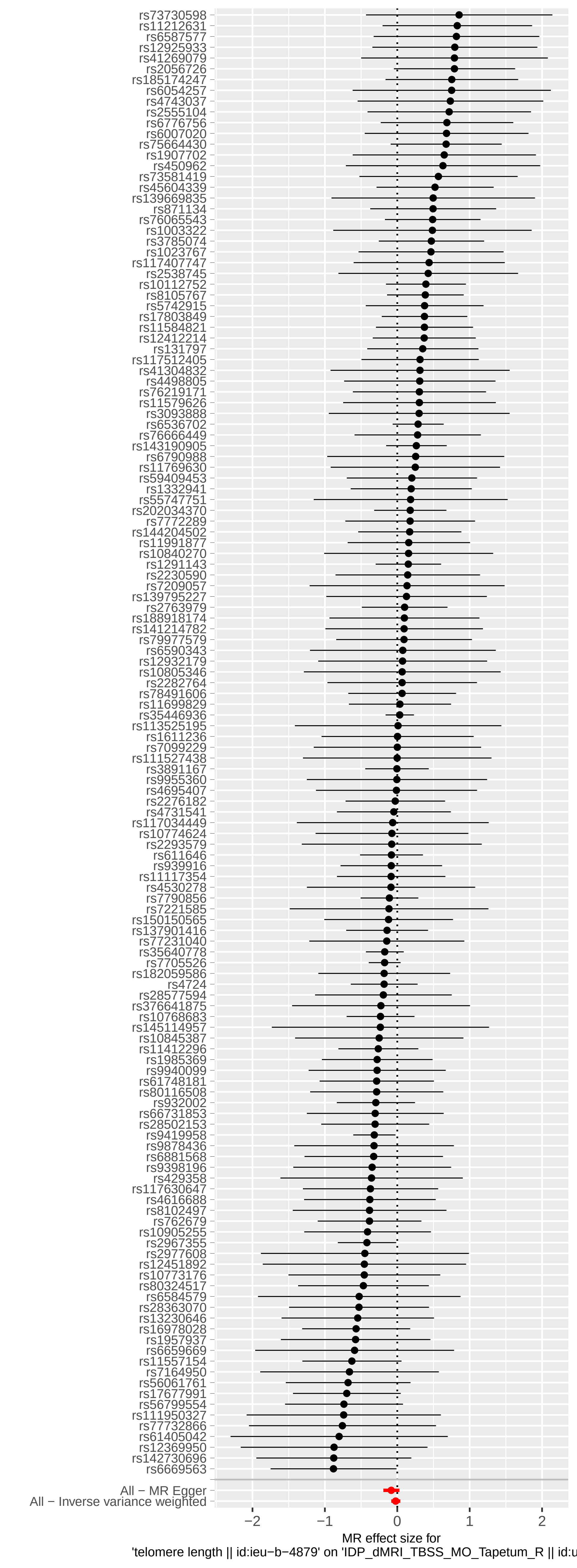
rs73730598
rs11212631
rs6587577
rs12925933
rs41269079
rs2056726
rs185174247
rs6054257
rs4743037
rs2555104
rs6776756
rs6007020
rs75664430
rs1907702
rs450962
rs73581419
rs45604339
rs139669835
rs871134
rs76065543
rs1003322
rs3785074
rs1023767
rs117407747
rs2538745
rs10112752
rs8105767
rs5742915
rs17803849
rs11584821
rs12412214
rs131797
rs117512405
rs41304832
rs4498805
rs76219171
rs11579626
rs3093888
rs6536702
rs76666449
rs143190905
rs6790988
rs11769630
rs59409453
rs1332941
rs55747751
rs202034370
rs7772289
rs144204502
rs11991877
rs10840270
rs1291143
rs2230590
rs7209057
rs139795227
rs2763979
rs188918174
rs141214782
rs79977579
rs6590343
rs12932179
rs10805346
rs2282764
rs78491606
rs11699829
rs35446936
rs113525195
rs1611236
rs7099229
rs111527438
rs3891167
rs9955360
rs4695407
rs2276182
rs4731541
rs117034449
rs10774624
rs2293579
rs611646
rs939916
rs11117354
rs4530278
rs7790856
rs7221585
rs150150565
rs137901416
rs77231040
rs35640778
rs7705526
rs182059586
rs4724
rs28577594
rs376641875
rs10768683
rs145114957
rs10845387
rs11412296
rs1985369
rs9940099
rs61748181
rs80116508
rs932002
rs66731853
rs28502153
rs9419958
rs9878436
rs6881568
rs9398196
rs429358
rs117630647
rs4616688
rs8102497
rs762679
rs10905255
rs2967355
rs2977608
rs12451892
rs10773176
rs80324517
rs6584579
rs28363070
rs13230646
rs16978028
rs1957937
rs6659669
rs11557154
rs7164950
rs56061761
rs17677991
rs56799554
rs111950327
rs77732866
rs61405042
rs12369950
rs142730696
rs6669563
All − MR Egger
All − Inverse variance weighted
−2
−1
0
1
2
MR effect size for
'telomere length || id:ieu−b−4879' on 'IDP_dMRI_TBSS_MO_Tapetum_R || id:u
